# Recent Advances in Ruthenium-Catalyzed Hydrogenation Reactions of Renewable Biomass-Derived Levulinic Acid in Aqueous Media

**DOI:** 10.3389/fchem.2020.00221

**Published:** 2020-04-21

**Authors:** Aristeidis Seretis, Perikleia Diamantopoulou, Ioanna Thanou, Panagiotis Tzevelekidis, Christos Fakas, Panagiotis Lilas, Georgios Papadogianakis

**Affiliations:** Industrial Chemistry Laboratory, Department of Chemistry, National and Kapodistrian University of Athens, Athens, Greece

**Keywords:** hydrogenation, renewable, platform chemical, levulinic acid, γ-valerolactone, water, biofuels, biorefinery

## Abstract

Levulinic acid (LA) is classified as a key platform chemical for the development of future biorefineries, owing to its broad spectrum of potential applications and because it is simply available from lignocellulosic biomass through inexpensive and high-yield production routes. Catalytic hydrogenation reactions of LA into the pivotal intermediate compound γ-valerolactone (GVL), and beyond GVL to yield valeric acid (VA), 1,4-pentanediol (1,4-PDO), and 2-methyltetrahydrofuran (2-MTHF) have gained considerable attention in the last decade. Among the various transition metals used as catalysts in LA hydrogenation reactions, ruthenium-based catalytic systems have been the most extensively applied by far, due to the inherent ability of ruthenium under mild conditions to hydrogenate the keto functionality of LA selectively into an alcohol group to form 4-hydroxyvaleric acid intermediate, which yields GVL spontaneously after dehydration and cyclization. This review focuses on recent advances in the field of aqueous-phase ruthenium-catalyzed hydrogenation reactions of LA toward GVL, VA, 1,4-PDO, 2-MTHF, 2-pentanol, and 2-butanol. It employs heterogeneous catalysts on solid supports, and heterogeneous water-dispersible catalytic nanoparticles or homogeneous water-soluble catalytic complexes with biphasic catalyst separation, for the *inter alia* production of advanced biofuels such as valeric biofuels and other classes of liquid transportation biofuels, value-added fine chemicals, solvents, additives to gasoline, and to food as well. The significance of the aqueous solvent to carry out catalytic hydrogenations of LA has been highlighted because the presence of water combines several advantages: (i) it is highly polar and thus an ideal medium to convert polar and hydrophilic substrates such as LA; (ii) water is involved as a byproduct; (iii) the presence of the aqueous solvent has a beneficial effect and enormously boosts hydrogenation rates. In sharp contrast, the use of various organic solvents gives rise to a dramatic drop in catalytic activities. The promotional effect of water was proven by numerous experimental investigations and several theoretical studies employing various types of catalytic systems; (iv) the large heat capacity of water renders it an excellent medium to perform large scale exothermic hydrogenations more safely and selectively; and (v) water is a non-toxic, safe, non-inflammable, abundantly available, ubiquitous, inexpensive, and green/sustainable solvent.

## Introduction

Currently, renewable biomass as a raw material is considered to be the grand challenge in the development of Green-Sustainable chemistry which decisively contributes to the transition from a fossil-based society to a bio-resources based economy. Biomass possesses a high potential as a raw material that sustainable biorefineries can use to manufacture biofuels, bio-based chemicals, solvents, materials, energy, power, pharmaceuticals, and food. The use of biomass as an industrial feedstock also combines environmental benefits because it mitigates local air pollution and the global warming problems caused by greenhouse gas emissions. Moreover, the use of biomass is associated with further economic, environmental, social, health and safety benefits which are based, *inter alia*, on the renewable nature of biomass, contrasted to the nature of fossil raw materials which possess a limited capacity in the domestic production of biofuels, and thus on the country's independence from fossil feedstocks, on the profits of local agriculture, on the implementations of financial incentives, and on biodegradability and biocompatibility, as well as on the mechanical and physicochemical properties of biomass-based products. The high potential of biomass is even more remarkable when one considers that the nature's global biomass production capacity is huge, namely about 2.0·10^11^ t/a compared to only 7·10^9^ t/a of all extracted fossil fuels, and furthermore that only 3.3% of the annual biomass production capacity is used for food, feed, and non-food applications (Van Bekkum and Gallezot, 2004; Sheldon, 2014; Li et al., 2018; Badgujar et al., 2020). Biomass consists of about 75% carbohydrates, 20% lignin, and 5% triglycerides, i.e., fats and oils, terpenes and proteins (Sheldon, 2014). Storage carbohydrates include the polysaccharides starch and inulin and the disaccharide sucrose. However, the most abundant biomass is lignocellulose, the constituent of plant cell walls, which is not edible and therefore without competition in food and consists of 40–50% cellulose, 25–35% hemicellulose, and 15–20% lignin (Climent et al., 2014). Cellulose is a linear biopolymer consisting entirely of glucose units linked by β-1,4-glycosidic bonds. The presence of extensive hydrogen bonding of both types inter- and intra-chain bonds imparts crystallinity to cellulose and strength to the structure of the plant, which makes cellulose recalcitrant to chemical or enzymatic hydrolysis. Cellulose is the organic compound with the largest capacity in the world. Hemicellulose is an amorphous branched biopolymer composed of C_5_ and C_6_ sugars with the major compound being xylose. Lignin is a cross-linked polyphenolic amorphous biopolymer surrounding hemicellulose and cellulose. The rigidity and complexity of the lignin structure gives recalcitrance to the cell walls of the plant, which makes difficult the pretreatment of lignocellulosic biomass for the liberation of cellulose and hemicellulose to be applied as feedstocks for the production of fuels, platform chemicals and materials.

**Figure 1:**
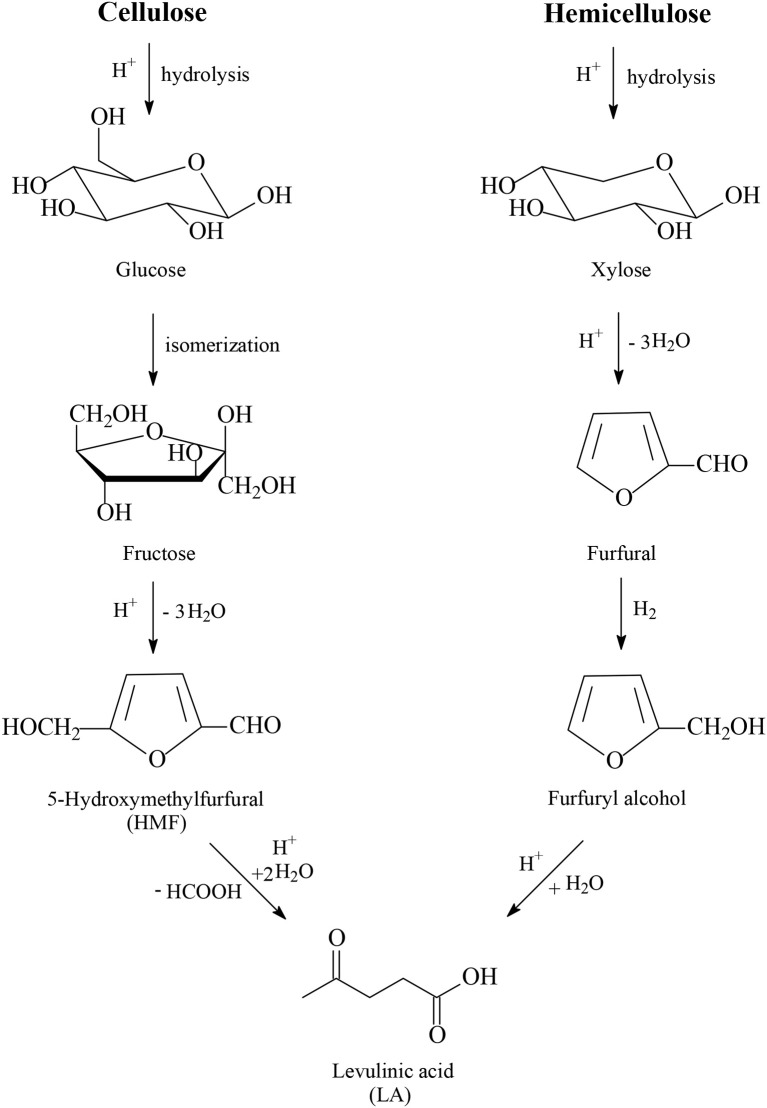
Production routes of levulinic acid from cellulose and hemicellulose.

One of the most promising platform chemicals obtained from lignocellulose is levulinic acid (LA, Figure 1), which was selected by the US Department of Energy (DOE) as one of the top twelve platform chemicals according to its report released in 2004 (Werpy and Petersen, 2004), as well as to the updated, revised and extended DOE's report in 2010 (Bozell and Petersen, 2010). LA is classified as a key platform chemical for the development of future biorefineries because of its broad spectrum of potential applications and because it is simply available with inexpensive and high yield production routes from lignocellulose biomass (Tang et al., 2014; Ye et al., 2020). The first industrial continuous process for the production of LA was developed by BioMetics Inc. in the earlies 1990's, which was well-known as the Biofine process, and proceeds in a proprietary two-reactor system using lignocellulose biomass as feedstock together with a sulfuric acid solution as a reagent. After the deconstruction of lignocellulose, sulfuric acid-catalyzed hydrolysis of cellulose and hemicellulose to yield C_6_ and C_5_ carbohydrates such as glucose and xylose takes place in the first reactor (Figure 1). Glucose is further isomerized into fructose and after dehydration of fructose the 5-hydroxymethylfurfural intermediate (HMF, Figure 1) is obtained under conditions to minimize polymerization side reactions, which yield undesired insoluble side products called humins. HMF is continuously removed and subsequently introduced as feedstock in the second reactor where, after hydration reactions, LA is produced with formic acid (Figure 1). The solid humins are separated from the solution of levulinic acid and burnt to generate heat and electricity for the process. From dehydration reactions of C_5_ carbohydrates e.g., xylose, furfural is produced. This is separately collected and acts as an intermediate after hydrogenation to furfuryl alcohol and hydration to also produce LA (Figure 1). The route of hemicellulose to LA possesses the advantage of milder acidic conditions and therefore of lower formation of undesired humins, because of the easier hydrolysis step of amorphous hemicellulose into C_5_ and C_6_ carbohydrates compared to the hydrolysis step of crystalline and recalcitrant cellulose into glucose intermediate, which is further isomerized into fructose and in the acid-catalyzed dehydration step to HMF unavoidable higher amounts of humins are obtained. The process engineering of Biofine was revised and redesigned by the GFBiochemicals company, which developed an acid catalyzed industrial process (Atlas Technology^TM^) for the manufacture of LA from lignocellulose biomass with a production capacity of 10,000 t/a. Several other industrial companies produce LA worldwide using lignocellulosic biomass as feedstock (Huber et al., 2006; Bozell and Petersen, 2010; Serrano-Ruiz et al., 2012; Bond et al., 2014; Climent et al., 2014; Leitner et al., 2017; Makhubela and Darkwa, 2018; Home page of GFBiochemicals company., 2019).

**Figure 2:**
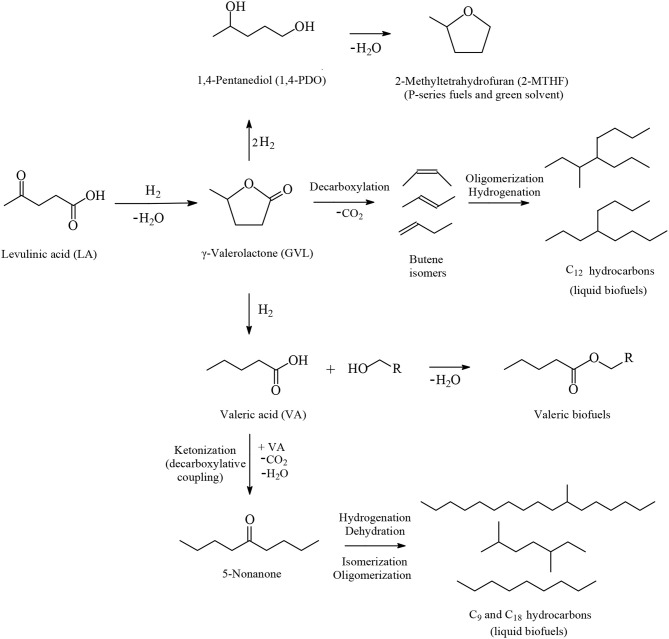
Routes based on the catalytic hydrogenation of LA to obtain advanced biofuels, chemicals and solvents.

Among the various routes for the valorization of LA, the catalytic hydrogenation route has been considered as an important pathway that is gaining more interest in recent years because of the wide range of potential applications of the LA's hydrogenation products, which include, *inter alia*, advanced biofuels such as valeric biofuels and other classes of liquid transportation biofuels, fine chemicals, solvents, additives to gasoline as well as additives to food. In the last decade, the hydrogenation reactions of LA into γ-valerolactone (GVL, Figure 2), which is a key intermediate compound, and beyond GVL to yield 1,4-pentanediol (1,4-PDO, Figure 2) and valeric acid (VA, Figure 2), have gained considerable attention. Numerous heterogeneous catalytic systems based on precious and non-noble metals and water-soluble transition metal catalytic complexes have been developed in the absence and presence of organic or aqueous solvents (Serrano-Ruiz et al., 2011, 2012; Tang et al., 2014; Yan et al., 2015a,b; Omoruyi et al., 2016; Pileidis and Titirici, 2016; Osatiashtiani et al., 2017; Makhubela and Darkwa, 2018; Xue et al., 2018; Dutta et al., 2019; Yu et al., 2019; Ye et al., 2020). The catalytic hydrogenation reaction of LA in the aqueous solvent is a more attractive and promising processing mode because the use of water combines several advantages: (i) the highly polar nature of the aqueous solvent makes it an ideal medium to convert polar, with high oxygen content, and hydrophilic platform chemicals such as the water-soluble starting material LA; (ii) water is involved as a byproduct to obtain the GVL intermediate, which is further hydrogenated into 1,4-PDO in the aqueous medium and, after dehydration and cyclization reactions 2-methyltetrahydrofuran (2-MTHF, Figure 2), is formed creating an aqueous/organic two-phase system which provides for the easy separation of the polar aqueous reaction medium from a polar organic product 2-MTHF by a simple phase separation. The same procedure could also be applied in the aqueous/organic biphasic system created with alkyl valerates obtained from LA hydrogenation product valeric acid (VA, Figure 2) after esterification with alcohols. This biphasic processing mode results in substantial energy savings, lower emissions and economic benefits; (iii) novel types of catalytic reactivities have been observed in water: in the hydrogenation reaction of LA into GVL, and beyond in the aqueous solvent employing conventional heterogeneous catalytic systems, catalytic nanoparticles, and water-soluble transition metal catalytic complexes, the presence of water has a beneficial effect and accelerates reaction rates, whereas in organic solvents much lower activities were observed. This promotional effect of water in the hydrogenation reaction of LA was proved by several experimental and theoretical studies using various types of catalytic systems; (iv) the large heat capacity of water makes it an excellent medium to perform exothermic reactions, such as the catalytic hydrogenation reaction of LA, more safely and selectively—which is especially important in large scale industrially-applied catalytic hydrogenation processes of LA; (v) water is a non-toxic, non-inflammable, abundantly available, inexpensive, ubiquitous, green and sustainable solvent.

In the LA hydrogenation reaction, ruthenium-based catalytic systems have been by far more extensively applied by than their catalytic counterparts based on other transition metals. This is because of the inherent and unique ability of ruthenium under mild reaction conditions to effectively hydrogenate the keto moiety of LA into an alcohol functionality to form the 4-hydroxyvaleric acid intermediate which spontaneously after dehydration and cyclization yields GVL. This review focuses on recent advances in the field of aqueous-phase ruthenium-catalyzed hydrogenation reactions of LA into GVL and beyond, to obtain VA, 1,4-PDO and 2-MTHF as well as 2-pentanol and 2-butanol employing heterogeneous catalytic systems on solid supports, heterogeneous water-dispersible catalytic nanoparticles and homogeneous water-soluble catalytic complexes for the production, *inter alia*, of advanced biofuels such as valeric biofuels and other classes of liquid transportation biofuels, value-added fine chemicals, solvents, additives to gasoline and to food as well. The significance of the aqueous solvent in such catalytic hydrogenation reactions has been also highlighted.

## Hydrogenation of LA Into Value-Added Chemicals and Advanced Biofuels

The catalytic hydrogenation reaction of LA yields GVL (Figure 2), which is an important C_5_ platform chemical and pivotal intermediate compound for the efficient conversion of LA to advanced biofuels of various classes, chemicals and solvents by several different routes as depicted in Figure 2. GVL is also used as an aprotic, polar, and sustainable solvent, as an additive to gasoline and suitable for use as a food additive. Hydrogenation and ring-opening reactions of GVL afford 1,4-PDO and VA intermediates (Figure 2). 1,4-PDO tends to undergo dehydration and cyclization reactions to yield 2-methyltetrahydrofuran (2-MTHF, Figure 2) which is considered as a green solvent (Yan et al., 2010, 2015a,b; Serrano-Ruiz et al., 2012) with a polarity placed between tetrahydrofuran and diethyl ether possessing, however, a potential to form explosive peroxides under air (Aycock, 2007; Fábos et al., 2009; Byrne et al., 2017). 2-MTHF is one of the three components of an alternative type of fuel, namely P-series fuels, that are approved by DOE as fuels that can substitute gasoline. Furthermore, 2-MTHF could be blended up to 70% in conventional gasoline fuels (Yan et al., 2010, 2015a,b; Serrano-Ruiz et al., 2012). Esterification reactions of VA with alcohols produce alkyl valerate advanced biofuels well-known as valeric biofuels, which are suitable to be blended with gasoline or diesel fuels depending on the length of the alkyl chain of added alcohol (Figure 2). When shorter chain alcohols such as methanol and ethanol are used in the esterification of VA the methyl and ethyl valerate products are suitable as gasoline fuel, whereas with longer chain alcohols such as butanol and pentanol, the butyl and pentyl valerates are more appropriate as diesel fuel with excellent energy density as well as volatility and ignition properties (Lange et al., 2010; Yan et al., 2010, 2015a,b; Serrano-Ruiz et al., 2012). VA could alternatively be used as starting material in decarboxylative coupling reactions i.e., ketonization reactions to produce 5-nonanone, which is an interesting platform to be applied as starting material in hydrogenation, dehydration, isomerization, and oligomerization reactions for the manufacture of C_9_ and C_18_ linear and branched hydrocarbons which are gasoline, diesel, and jet biofuels (Figure 2) (Serrano-Ruiz et al., 2012; Simakova and Murzin, 2016). Another route for the production of jet biofuels is based on GVL, which in the first step undergoes decarboxylation into butenes, followed by oligomerization to form higher alkenes which after hydrogenation are suitable as aviation biofuels (Figure 2) (Bond et al., 2010, 2014).

### Hydrogenation of LA Into GVL

**Figure 3:**
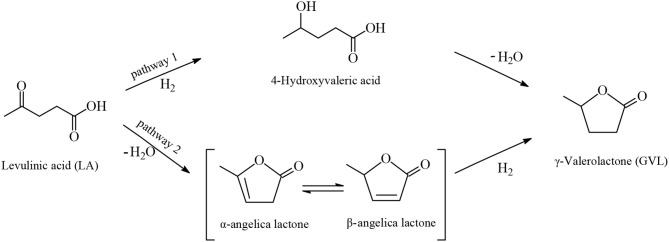
Possible reaction mechanisms for the synthesis of GVL from LA.

**Figure 4:**
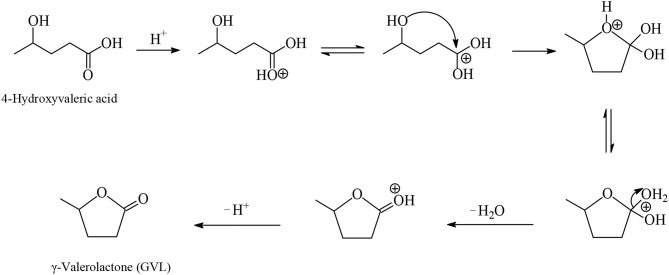
Proposed mechanism for the dehydration of 4-hydroxyvaleric acid intermediate to yield by intramolecular esterification GVL (Ruppert et al., 2015).

GVL is a water-soluble, non-toxic liquid that is stable at neutral pH in aqueous solvent and air without any formation of a measurable amount of peroxides when in a glass flask exposed to the air for several weeks, making it safe for industrial scale use. Furthermore, GVL does not form an azeotropic mixture with the aqueous solvent and therefore the manufacture of GVL constitutes a less energy demanding process compared to that for the production of absolute ethanol. Two possible reaction pathways were proposed for the synthesis of GVL from LA which are shown in Figure 3. According to the first pathway the keto functionality of LA is hydrogenated probably by heterolytic H_2_ cleavage into an alcohol group to form the 4-hydroxyvaleric acid intermediate, which then readily undergoes dehydration to give, by favorable intramolecular esterification, the cyclic ester product GVL (Figures 3, 4). The heterolytic dihydrogen cleavage mechanism may be facilitated by heterogenous catalytic systems comprising a transition metal and an oxide as support, such as Ru/TiO_2_, Ru/Al_2_O_3_, by the interaction between the metal with the support in the way that on the surface of transition metal is formed the H^−^ moiety of dihydrogen and on the oxygen atom of the support the H^+^ moiety (Kubas, 2005). The second possible pathway of Figure 3 involves acid-catalyzed endothermic dehydration of LA at reaction temperatures higher than 180°C to form α-angelica lactone by intramolecular esterification, which is subsequently hydrogenated to yield GVL. In both mechanisms depicted in Figure 3 the hydrogenation steps are influenced by the nature and activity of the transition metal of the catalysts, whereas the dehydration steps depend on the acidity of the catalysts and of the medium. At elevated temperatures and/or in the presence of acidic heterogeneous catalytic systems the dehydration reaction of LA is promoted to give α-angelica lactone, which easily polymerizes on acidic surfaces to form coke, resulting in a severe deactivation of the heterogeneous catalytic system.

#### Heterogeneous Catalysts on Solid Supports

Numerous heterogeneous ruthenium-based catalytic systems have been successfully applied in the hydrogenation reaction of LA into GVL usually employing relatively more forcing conditions, i.e., higher temperatures and dihydrogen pressures in aqueous media. The use of heterogeneous catalysts combines the easy catalyst separation from reaction products and catalyst recycling with the possibility to operate easily in a continuous process.

**Figure 5:**
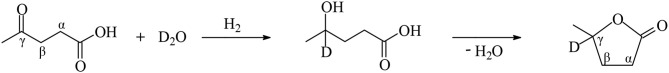
Hydrogenation reaction of LA in D_2_O.

Tan et al. (2015) synthesized ruthenium-supported catalysts with low 0.5 wt.%, moderate 1 wt.% and higher 2 wt.% ruthenium loading on various oxides such as Al_2_*O*_3_, SiO_2_, ZrO_2_, and TiO_2_ and investigated their catalytic activity and selectivity on the hydrogenation reaction of LA into GVL at molar ratios of LA/Ru between 8,707 and 2,177, reaction temperatures of 130 and 70°C under 40 bar of dihydrogen pressure in aqueous and organic media. With a higher ruthenium loading of 2 wt.% all supported catalysts at 130°C exhibited a TOF value of 4,353 per hour with quantitative conversions of LA. At a low ruthenium loading of 0.5 wt.% the Ru/TiO_2_ catalytic system exhibited at 130°C within 3 h reaction time the highest catalytic activity of TOF = 2,769 h^−1^ with a conversion of LA of 95.4 mol%, compared with the other ruthenium-supported catalysts under the same conditions which have shown lower activities with e.g., 0.5 wt.% Ru/Al_2_*O*_3_ the catalytic activity has been dropped down to 401 TOF's per hour and the conversion of LA to 13.8 mol% (Table 1). A much higher catalytic activity of TOF = 7,662 h^−1^ has been obtained with Ru/TiO_2_ catalysts with a moderate ruthenium loading of 1 wt.% even at a lower temperature of 70°C within a 15 min reaction time in the aqueous solvent. The higher catalytic activity of Ru/TiO_2_ could be explained by both the small size of ruthenium nanoparticles and the better dispersion of the metal on the support, which was proven by TEM analysis in showing average size of ruthenium nanoparticles on the TiO_2_ support were smaller, namely 2.0 nm, and more uniform compared with their counterparts on the ZrO_2_ support. Furthermore, the nature of the support plays a crucial role in the heterogeneously ruthenium-catalyzed LA hydrogenation reaction, because the support activates the C=O functionality of LA and ruthenium nanoparticles dissociates dihydrogen. It is highly probable that a strong interaction between small ruthenium particles and TiO_2_ support takes place which, after activation of the carbonyl moiety on TiO_2_, generates highly active sites on the coordination sphere of the metal by catalytic relevant ruthenium species for efficient H_2_ dissociation. With all ruthenium-supported catalysts under various reaction conditions in aqueous medium, the only product obtained was GVL with essentially quantitative selectivity. A remarkable solvent effect has been observed in the LA hydrogenation reaction with 1 wt.% Ru/TiO_2_ catalysts at 130°C and 40 bar in the presence of aqueous and organic solvents. When water was used as a solvent in the LA hydrogenation the catalytic activity was exceptionally high and reached a value of TOF = 8,707 h^−1^, whereas with organic solvents such as ethanol and 1,4-dioxane, the catalytic activity dramatically drops down to 3,261 and 1,337 TOF's per hour, respectively, under the same reaction conditions (Table 1). The promotional effect of water was rationalized by assuming that water acts not only as a solvent but also participates as a reactant after dissociation in this aqueous-phase catalytic hydrogenation reaction. To verify this assumption, Tan et al. carried out the LA hydrogenation reaction in D_2_O and found by NMR and GC/MS analysis of the products that a D atom was bonded to the γ-C atom of the GVL product, which originates not from the dihydrogen reactant but from D_2_O solvent (Figure 5). Furthermore, the authors mixed GVL with D_2_O without a D atom to be detected on γ-C atom of GVL, and ruled out an alternative pathway of the incorporation of the D atom in GVL during the hydrogenation reaction of LA in D_2_O,

**Table 1:** Ruthenium-based heterogeneous catalysts on solid supports and water-dispersible catalytic nanoparticles for the hydrogenation of LA toward GVL.

**Catalysts**	**Molar** **ratio** **LA/M**	**T** **(^**°**^C)**	**P_**H2**_** **(bar)**	**t** **(h)**	**Solvent**	**Conversion LA** **(mol%)**	**Selectivity GVL** **(mol%)**	**TOF^a^** **(h^−1^)**	**References**
0.5% Ru/Al_2_*O*_3_	8,707	130	40	3	Water	13.8	99.9	401	Tan et al., 2015
0.5% Ru/SiO_2_	8,707	130	40	3	Water	80.1	99.8	2,325	
0.5% Ru/ZrO_2_	8,707	130	40	3	Water	80.3	99.9	2,331	
0.5% Ru/TiO_2_	8,707	130	40	3	Water	95.4	99.9	2,769	
1% Ru/TiO_2_	4,353	70	40	0.25	Water	44	99.9	7,662	
1% Ru/TiO_2_	4,353	130	40	0.5	Water	100	99.9	8,707	
1% Ru/TiO_2_	4,353	130	40	1	Ethanol	74.9	47.7	3,261	
1% Ru/TiO_2_	4,353	130	40	1	1,4-Dioxane	30.7	99.9	1,337	
1% Ru/OMS	3,831	100	30	1	Water	99.9	99.8	3,420	Molleti et al., 2018
RuCl_3_·3H_2_O/TiO_2_	4,350	90	45	4	Water	86	92	1,152	Piskun et al., 2018
RuNO(NO_3_)_3_/TiO_2_	4,350	90	45	4	Water	77	94	824	
5% Ru/ZrO_2_	2,100	170	27	7	Water	99.0	99.9	297	Filiz et al., 2017
2% Ru/FLG	1,460	20	40	8	Water	99.3	97.7	184	Xiao et al., 2016
5% Ru/C	359	130	12	2.7	Water	99.5	86.6	133	Al-Shaal et al., 2012
0.64% Ru/TiO_2_	247	150	35	5	Water	100	93	49	Primo et al., 2011
5% Ru/TiO_2_	106	150	35	5	Water	100	90	21	
0.83% Ru/TiO_2_ ultrathin	4,878	100	40	1.5	Water	100	99.1	19,045^b^	Gao et al., 2019
Ru/NCS	10,000	70	40	1	Water	51	100	9,858	Liu et al., 2019
Ru/CS	10,000	70	40	1	Water	32	100	6,985	
Ru/TiO_2_	505	150	20	5	Water	100	94.8^c^	101	Ndolomingo and Meijboom, 2019
RuCl_3_·3H_2_O/PEG400	40	130	20	1	PEG/Water	99	99	40	Patil and Bhanage, 2016
Ru_3_(CO)_12_	1,720	130	5	12	Water	100	100	143	Ortiz-Cervantes and García, 2013
RuNHC	1,000	130	12	2.7	Water	99	96	361	Tay et al., 2016
RuNHC	1,000	130	12	2.7	THF	1	1	3	
Ru@PEG-CD	1,300	80	40	4	Water	97	99	315	Chen et al., 2017
Ru@PVP	1,300	80	40	4	Water	93	99	302	

a *Defined as mole of hydrogenated LA per mole of ruthenium per hour*.

b *Defined as mole of hydrogenated LA per number of surface ruthenium atoms per hour*.

c *Selectivity toward 2-MTHF: 5.2 mol%*.

which might be due to an H/D exchange between D_2_O and GVL. Thus, is proven that the promotional effect of the aqueous solvent in the LA hydrogenation is based on the involvement of water after dissociation as a reactant in the mechanism of this aqueous-phase catalytic reaction. Michel et al. (2014) applied Ru/TiO_2_, Pt/TiO_2_, and Pd/TiO_2_ catalysts possessing a similar metal particle size between 2.1 and 3.2 nm with an uniform distribution of each metal on the titania support in the LA hydrogenation reaction to GVL, under mild reaction conditions of 70°C and 50 bar dihydrogen pressure, using water and tetrahydrofuran as solvents. The catalytic activity of Ru/TiO_2_ is strongly influenced by the nature of reaction solvent. In the aqueous medium Ru/TiO_2_ catalysts exhibited the highest catalytic activity to obtain essentially quantitative conversions of LA with a yield of 95 mol% of GVL, whereas in organic solvents such as tetrahydrofuran the Ru/TiO_2_ system shows no catalytic activity in this hydrogenation reaction. In sharp contrast, the solvent did not influence the catalytic activities of Pt/TiO_2_ and Pd/TiO_2_ to obtain with Pt/TiO_2_ catalysts in both aqueous and organic medium low conversions of LA in the range of 20 up to 30 mol % and yields of GVL between 15 and 20 mol%. A very low catalytic activity has been observed with Pd/TiO_2_ catalysts under these mild reaction conditions. To explain the obtained experimental results, the authors performed Density Functional Theory (DFT) calculations using acetone as a model substrate containing a C=O functionality chemisorbed on Ru(0001) surface, and proved that the nature of the aqueous solvent is the reason for the higher activity of Ru/TiO_2_ catalysts in water, because the hydrogen bond of a chemisorbed water molecule on Ru-surface formed with the oxygen atom of an adjacent chemisorbed alkoxy intermediate involved in the reaction mechanism enormously lowers the activation energy barrier of the reaction pathway, resulting in enhanced reaction rates among Ru/TiO_2_-catalyzed hydrogenation reactions in aqueous media. It should be mentioned that in these DFT calculations the higher activity of ruthenium catalysts in the hydrogenation of acetone in aqueous media was attributed to the formation of hydrogen bonds between chemisorbed water and carbonyl intermediate compounds, and in these calculations the possibility that water may also act as a reactant after dissociation on the ruthenium surface has not been considered. Michel and Gallezot (2015) pointed out that in order to be able to understand the high catalytic activity of ruthenium compared with other transition metals in hydrogenation reactions of starting materials bearing C=O functionalities in water where two mechanisms are operative (the first one includes chemisorbed water molecules which, by hydrogen bonding, lower the energetic span of the reaction pathway to yield the –CH_2_OH functionality by dissociation of hydrogen, and the second one involves dissociation of water to produce hydrogen atoms to participate in the hydrogenation reaction of the C=O group into a –CH_2_OH moiety), it is necessary to carry out more isotope labeling experiments and also to perform further improved theoretical investigations including both of these mechanisms. Continuous aqueous-phase hydrogenation reactions of LA in a fixed-bed reactor have been carried out over Ru/C, Ru/Al_2_*O*_3_ and Ru/MgO catalysts at a high temperature of 275°C under atmospheric pressure of dihydrogen, and the results revealed that Ru/C catalysts exhibited higher conversion of LA with higher selectivity toward GVL compared with Ru/Al_2_*O*_3_ and Ru/MgO catalytic systems (Velisoju et al., 2018). The authors further investigated the role of water on the activity over Ru/C catalysts in the continuous hydrogenation of LA toward GVL using various concentrations of LA in water from 70 wt.% down to 5 wt.%, and found that with increasing amounts of water increased both the conversion of LA and the selectivity to GVL, employing probe-adsorbed diffuse reflectance infrared Fourier transform (DRIFT) spectroscopy. Velisoju et al. found that -OH groups were generated in the presence of water on the surface of ruthenium of Ru/C catalyst. According to their proposed mechanism such hydroxyl groups initiate the dehydration step of the enol form of LA to yield α-angelica lactone intermediate, and after isomerization to β-angelica lactone surface ruthenium metal hydride species are responsible for the hydrogenation step of the -C=C- functionality of β-angelica lactone to form GVL (Figure 6). Therefore, the higher catalytic activity of Ru/C catalysts in the presence of water in the vapor phase is because of the easier generation of –OH groups on the surface of ruthenium, and also due to the presence of a larger active ruthenium metal surface area in comparison to the other Ru/Al_2_*O*_3_ and Ru/MgO catalysts. Mamun et al. (2019) studied solvent effects on the reaction kinetics of the hydrogenation reaction of LA into GVL over Ru(0001) catalysts under various conditions, using three media of different polarity—namely water, methanol, and 1,4-dioxane. The presence of the aqueous solvent enormously facilitated the hydrogenation reaction kinetics and this solvent effect is much stronger at temperatures lower than 100°C. For example, at the low reaction temperature of 50°C, a rate increase of 4 orders of magnitude was obtained due to the solvation effect of water. The strongest solvent effect has been observed in the highly polar solvent water and this effect decreases with decreasing polarity of the solvent, i.e., in the order: water > methanol > 1,4-dioxane. Various transition metals such as ruthenium, palladium, platinum, and nickel supported on hydroxyapatite (HAP) have been applied as catalysts in the liquid phase hydrogenation reaction of LA to GVL at 70°C under 5 bar H_2_ pressure in various solvents such as water, ethanol, and toluene (Sudhakar et al., 2014). The highest catalytic activity exhibited ruthenium catalysts and the rates decrease in the order: Ru > Pt > Pd > Ni. With Ru/HAP catalysts the highest activity has been found to be in the aqueous solvent and the catalytic activity decreased with decreasing polarity of the solvent: water > ethanol > toluene. Sudhakar et al. (2016) further examined the activity of ruthenium, palladium, platinum copper, and nickel catalysts supported on HAP in the vapor phase hydrogenation of LA in the presence of water. The obtained rates with such catalysts have the following order: Ru > Pt > Cu > Pd > Ni. At the reaction temperature of 275°C in the Ru/HAP-catalyzed hydrogenation in the presence of water the LA conversion was 65.1 mol% and the selectivity toward GVL 99.8 mol%. In sharp contrast in the absence of water under the same conditions the conversion of LA gives rise to a dramatic drop to 4.0 mol%, and the only product observed was α-angelica lactone. At the higher temperature of 425°C in the presence of water with Ru/HAP catalysts, the conversion of LA increased to 94.0 mol% with the selectivity to GVL to decrease to 80.5 mol% with concomitant formation of α-angelica lactone (13.0 mol%) and β-angelica lactone (5.5 mol%). Ru/HAP catalysts showed a better activity in the presence of water in comparison to organic solvents such as ethanol and methanol. Gundekari and Srinivasan (2019) prepared α novel hydrous ruthenium oxide (HRO) catalyst precursor by a precipitation method using an aqueous solution of RuCl_3_·3*H*_2_O with CaCO_3_ and applied HRO systems in the hydrogenation reaction of LA under mild conditions of 50°C and 10 bar dihydrogen pressure to obtain quantitative conversions of LA and yields toward GVL within 30 min in the aqueous solvent. The HRO precursor which possess water molecules strongly bonded to RuO_x_ surface was reduced to catalytic active Ru(0) species under hydrogenation reaction conditions. Quantitative conversions of LA by HRO catalysts precursors were obtained only in the presence of the aqueous solvent, whereas in organic solvents such as THF and methanol the conversions of LA were very low, i.e., between 2 and 5 mol% at 100°C, 10 bar dihydrogen within 15 min reaction time. HRO catalyst precursors supported on H-β zeolite could be recycled in five consecutive runs with the Ru-HRO/H-β catalyst to retain its activity. Guo et al. (2016) investigated the catalytic properties of Ru@MIL-101(Cr) and Ru@MIL-100(Cr) Metal-Organic Frameworks as well as of Ru@HY-zeolite (Si/Al ≥ 5.2) in the hydrogenation of LA into GVL in aqueous media. The highest catalytic activity was exhibited by the Ru@MIL-101(Cr) system to obtain quantitative conversion of LA with an essentially quantitative selectivity toward GVL under mild reaction conditions at 70°C under 10 bar dihydrogen pressure within 5 h reaction time in water. The authors investigated the catalytic behavior of Ru@MIL-101(Cr) in the presence of water and various organic solvents. The strongest solvent effect, i.e., the highest catalytic activity, has been observed in the highly polar aqueous solvent. This effect decreases with decreasing polarity of the solvent, in the order: water > methanol > ethanol > 2-propanol > 1,4-dioxane. Ultrafinely dispersed ruthenium nanoparticles with a mean size of 2.1 nm have been supported on N-doped hierarchically-porous carbon (Ru/NHPC) with the support to be prepared from cellulose, and applied as catalysts in the aqueous-phase hydrogenation of LA to obtain higher activities, selectivity toward GVL, and (especially) stabilities compared with conventional Ru/C catalytic systems (Wei et al., 2018). Recycling experiments have shown that the Ru/NHPC catalytic system could be reused in thirteen consecutive hydrogenation runs at 50°C, 10 bar H_2_ pressure within 3 h in aqueous media without any obvious deactivation, whereas the Ru/C catalyst under identical conditions was severely deactivated already in the 3rd recycling consecutive run. The authors further compared the catalytic activity of Ru/NHPC systems in water and different organic solvents such as DMF, 1,4-dioxane, cyclohexane, methanol, and ethanol, and found that the highest yields toward GVL were obtained in water in comparison to all used organic solvents.

**Figure 6:**
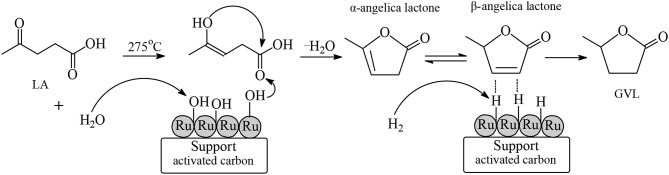
Proposed mechanism for the hydrogenation of LA to GVL at 275°C on the surface of Ru/C catalyst in aqueous media.

**Figure 7:**
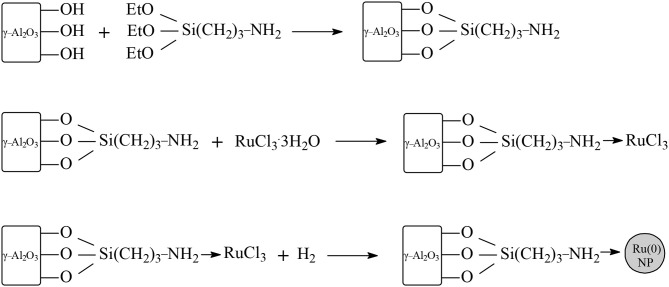
Synthesis of the Ru/NH_2_-γ-Al_2_*O*_3_ catalytic system.

In studies of ruthenium-based catalytic systems a main focus is on the supports, with the aim to improve the efficiency of the catalyst via pre-treatment methods, modification of the nature and control of the properties, and the structure of the support material. Novel ruthenium supported on manganese oxide octahedral molecular sieve (Ru/OMS) systems have been synthesized and applied as catalysts in the hydrogenation of LA at 100°C under 30 bar dihydrogen pressure and a molar ratio of LA/Ru = 3,831 within 1 h in aqueous media (Molleti et al., 2018). Under these conditions the catalytic activity exhibited Ru/OMS catalyst was high (TOF = 3,420 h^−1^) to give near quantitative conversion of LA and selectivity toward GVL with retained catalytic activity in four recycling consecutive experiments (Table 1). This high catalytic activity exhibited Ru/OMS catalysts could be explained by the moderate acidity of the novel supported system and the high dispersion of ruthenium nanoparticles on the surface. The apparent activation energy of Ru/OMS catalyst in the aqueous-phase hydrogenation of LA was calculated and amounts to only 49.16 kJ/mol. Piskun et al. (2018) have shown that the performance of Ru/TiO_2_ (anatase) catalysts applied in the aqueous-phase hydrogenation of LA to GVL strongly depends on the synthesis protocol of the catalytic system by varying the nature of the ruthenium precursor, the calcination and/or reduction step, and the amount of dihydrogen in the gas during the reduction step. The highest initial reaction rates in the aqueous-phase hydrogenation of LA to GVL at 90°C and 45 bar dihydrogen pressure were obtained with RuCl_3_·3H_2_O catalysts precursors compared to RuNO(NO_3_)_3_ precursors (Table 1) without an intermediate calcination step using only 10% H_2_ in the reduction gas, whereas a calcination step and the presence of a H_2_ rich reduction gas gives rise to a drop in catalytic activity. The influence of different phases of titania supports, i.e., rutile and anatase on ruthenium and platinum catalysts, has been investigated in the hydrogenation of LA into GVL at 30°C or 70°C under 50 bar of H_2_ pressure in aqueous media (Ruppert et al., 2015). It has been surprisingly found that the influence of the type of support was not the same for ruthenium and platinum catalysts. The highest catalytic activity was exhibited by ruthenium on TiO_2_ support, consisting of 10% rutile and 90% anatase to obtain at 70°C quantitative conversion of LA and yield to GVL within 1 h, whereas with Ru on TiO_2_ support consisting of 100% anatase the conversion of LA was only 38 mol% and the yield to GVL of only 31 mol% under the same conditions. Microscopy studies showed that the ruthenium particles were exclusively located on the less rutile crystallites and could be understood due to a higher adhesion of ruthenium on rutile compared to anatase. In contrast, with platinum on TiO_2_ support consisting of 10% rutile and 90% anatase, the conversion of LA was 22 mol% and the yield to GVL of 18 mol%, whereas a higher conversion of LA of 54 mol% and a higher yield to GVL of 50 mol% were obtained by platinum catalysts on TiO_2_ support consisting of 100% of anatase. Novel Ru/TiO_2_ systems have been synthesized by photoassisted controlled modification of the TiO_2_ support by addition of calcium and applied as catalysts in the transfer hydrogenation of LA using formic acid as an internal hydrogen source in aqueous media (Wojciechowska et al., 2019). The Ca-modification of Ru/TiO_2_ catalysts enhanced the performance of both catalytic steps, the transfer hydrogenation of LA into GVL, and also the dehydrogenation of formic acid to yield the dihydrogen reactant. The Ca-modification caused both a decreased size of the anatase crystallite and the formation of a new phase of calcium titanate, resulting in smaller ruthenium metal particles stabilized on the support. The superior catalytic activity of Ca-modified Ru/TiO_2_ systems could be further understood probably due to the strong interaction between the smaller ruthenium particles with the Ca-modified TiO_2_ support. Gao et al. (2018) disclosed results on the stability of 2.5 wt.% Ru/ZrO_2_ catalysts applied in the transfer hydrogenation reaction of LA to GVL with a mixture of formic acid/formate at 150°C in aqueous media, and found that the incorporation of 0.1 wt.% SiO_2_ to the support enormously stabilized the system and the catalyst Ru/ZrO_2_-SiO_2_ retained its activity after recycling in three consecutive runs, whereas the activity of Ru/ZrO_2_ catalyst significantly decreased after recycling already in the first consecutive run. The catalytic properties of four different Ru/ZrO_2_ systems have been evaluated in the hydrogenation of LA to GVL in aqueous media; catalytic activity is strongly influenced by the pre-treatment method and the most active catalyst was obtained after impregnation of RuCl_3_·3H_2_O on pre-calcined zirconia support, followed by reduction with dihydrogen to give ruthenium metal particles of a size between 3.0 and 4.0 nm with an uniform dispersion (Filiz et al., 2017). Using this Ru/ZrO_2_ catalyst in the aqueous phase hydrogenation of LA at 170°C under 27 bar of H_2_ within 7 h at a molar ratio of LA/Ru = 2100, the catalytic activity obtained was 297 TOFs per hour with a conversion of LA of 99.0 mol% and a selectivity toward GVL of 99.9 mol% (Table 1). Stability studies of Ru/TiO_2_, Ru/ZrO_2_, and Ru/C catalytic systems have been reported on the hydrogenation reaction of LA to GVL at 150°C under 30 bar dihydrogen pressure in 1,4-dioxane and found that the Ru/TiO_2_ benchmark catalyst was deactivated just after the first recycling run, whereas the Ru/ZrO_2_ catalyst maintained its activity even after five recycling consecutive runs (Ftouni et al., 2016). The authors have clearly shown that the presence of the aqueous solvent has a promotional effect in the Ru/ZrO_2_-catalyzed hydrogenation reaction of LA to GVL. Thus, in a solvent mixture of 1 wt.% water with 99 wt.% 1,4-dioxane an increase in the GVL yield of 25 mol% was observed compared to anhydrous 1,4-dioxane solvent and in a mixture of 10 wt.% water with 90 wt.% 1,4-dioxane the catalytic activity further increased to obtain quantitative GVL yields. Ren et al. (2019) studied a cascade process for the direct conversion of carbohydrates fructose and glucose, and of storage or structural polysaccharides starch or cellulose toward GVL catalyzed by a system comprising the heteropoly acid-based ionic liquid 1-methyl-3-(3-sulfopropylimidazolium) silicotungstate and Ru/ZrO_2_ in aqueous media. Using this catalytic system at 180°C under argon for 3 h for the dehydration step and then under 40 bar dihydrogen pressure within 10 h for the hydrogenation step, the GVL yields obtained from fructose, glucose, starch, and cellulose were 63, 68, 60, and 60mol%, respectively. Recycling experiments with four consecutive runs have shown deactivation of Ru/ZrO_2_ catalyst component due to massive coke deposition formed probably from decomposition of humins and α-angelica lactone obtained as side products and intermediates at the reaction temperature of 180°C under acidic conditions. Raspolli Galletti et al. (2013) investigated the one pot approach for the conversion of giant reed (*Arundo donax L*.) directly to GVL by a combination process of acid catalyzed dehydration and catalytic hydrogenation reactions employing a bifunctional catalyst comprising of Ru/C and niobium phosphate or niobium oxide at a low temperature of 70°C and low dihydrogen pressure, i.e., 5 bar, to yield up to 16.6 mol% GVL, based on dry biomass with an essentially quantitative conversion of the LA intermediate in aqueous media. Xiao et al. (2016) synthesized few-layered graphene (FLG)-supported ruthenium nanoparticles and applied as them catalysts in the hydrogenation of LA in aqueous media to obtain at room temperature under 40 bar of H_2_ within 8 h a conversion of LA of 99.3 mol% and selectivity toward GVL of 97.7 mol% (Table 1). In five recycling experiments the 2.0% Ru/FLG catalyst retained its activity and exhibited four times higher activity compared with ruthenium catalysts on conventional support of activated carbon (Ru/C). Various ruthenium supported catalysts such as Ru/C, Ru/SiO_2_, and Ru/Al_2_*O*_3_ have been screened in the hydrogenation of LA into GVL under mild reaction conditions (25°C, 12 bar H_2_) in the absence of solvents with the Ru/C catalyst to show the highest catalytic activity, whereas Ru/SiO_2_ and Ru/Al_2_*O*_3_ exhibited significantly lower yields toward GVL (Al-Shaal et al., 2012). The commercial 5 wt.% Ru/C catalytic system has been further applied in the aqueous-phase hydrogenation of LA at 130°C under 12 bar of dihydrogen pressure within 2.7 h reaction time, and exhibited a moderate catalytic activity of 133 TOFs per hour (Table 1). It should be mentioned that conventional Ru/C catalysts suffer from enormous deactivation even at low temperatures and pressures, probably because of the suppressed mass transport of LA through the small-sized micropores resulting in a pore blocking of the carbon material. Primo et al. (2011) investigated the activity of 5 wt.% Ru/TiO_2_ and 0.64 wt.% Ru/TiO_2_ catalysts in the aqueous-phase hydrogenation of LA into GVL and found that the activity of 0.64 wt.% Ru/TiO_2_ is about two times higher compared with the activity of 5 wt.% Ru/TiO_2_ catalyst (Table 1), and could be explained due to the synergism between the small size of ruthenium nanoparticles and the activation of the carbonyl functionality of LA on the TiO_2_ support. Highly active ruthenium nanoparticles of a mean size of 1.4 nm supported on ultrathin TiO_2_ nanosheets with an unique two dimensional (2D) structure have been applied as catalysts in the hydrogenation of LA toward GVL (Table 1) to obtain impressively higher intrinsic catalytic activities (TOF = 19,045 h^−1^) under relative mild reaction conditions of 100°C and 40 bar dihydrogen pressure within 90 min reaction time, compared with those of conventional Ru/SiO_2_, Ru/MoS_2_, and Ru/C catalytic systems, which exhibited 7,089, 693, and 1,909 TOFs per hour, respectively, under identical conditions in aqueous media. It is relevant to point out that the calculation of TOF values in this work (Table 1) is based on surface ruthenium atoms and not on the bulk ruthenium loading (Gao et al., 2019). Recycling experiments have shown that the Ru/TiO_2_ ultrathin nanosheets catalyst could be reused in six consecutive hydrogenation runs without any deactivation. The apparent activation energy of the Ru/TiO_2_ ultrathin nanosheets catalyst in the aqueous-phase hydrogenation of LA was calculated and amounts to only 43.4 kJ/mol, which is much lower than that of Ru/C catalyst (E_app_= 87.66 kJ/mol), indicating a different nature of catalytically active sites. Employing high-resolution transmission electron microscopy (HRTEM), X-ray photoelectron spectroscopy (XPS), and temperature programmed reduction of hydrogen (H_2_-TPR) techniques has been revealed that in the Ru/TiO_2_ ultrathin nanosheets catalyst the ruthenium atoms are chemically bonded to oxygen atoms of TiO_2_ support to form Ru-O-Ti bonds, which cause a strong interfacial interaction. Whereas, in Ru/SiO_2_ and Ru/MoS_2_ catalysts the ruthenium atoms interact weakly with their supports, although a strong coordination has been observed between each ruthenium with their neighboring ruthenium atoms. The authors performed DFT calculations using a Ru/TiO_2_ modeled interface or a model of the most exposed Ru (0002) surface for the hydrogenation of LA, and have shown that the preferential mechanistic pathway is hydrogenation of the keto functionality with formation of ${\text{CH}}_{3}CH\left(O\right)CH_{2}CH_{2}COOH^{*}$, cyclization to GVL-OH and dehydroxylation to give GVL, independent of the structure of the surface. Interestingly, with the Ru/TiO_2_ interfacial structure, the rate determining step changes from hydrogenation to cyclization compared to Ru (002) surface, where the rate determining step is the hydrogenation one. In addition, to rationalize the results of exceptionally high catalytic activities exhibited by Ru/TiO_2_ ultrathin nanosheets catalysts where a strong interfacial interaction takes place due to the presence of Ru-O-Ti chemical bonds, we assume that such a system enormously facilitates both the heterolytic dihydrogen cleavage mechanism necessary for the easy hydrogenation of the keto functionality of LA into an alcohol group to yield the 4-hydroxyvaleric acid intermediate (*vide supra*, Unit 2.1 Hydrogenation of LA into GVL) and the dehydration step, as well to yield GVL. Van Nguyen et al. (2019) prepared via *de novo* synthesis a RuCl_3_@MIL-53-NH_2_ Metal-Organic Framework which was reduced at 500°C under H_2_/N_2_ gas flow to obtain the Ru@C-Al_2_*O*_3_ catalyst which consists of ruthenium nanoparticles confined in the C-Al_2_*O*_3_ honeycomb system. Ru@C-Al_2_*O*_3_ catalysts have been applied in the hydrogenation of LA under very mild conditions, i.e., at room temperature and atmospheric pressure of dihydrogen, to obtain a quantitative conversion of LA and a GVL yield of 99.9 mol% within 6 h of reaction time in the aqueous medium. Recycling experiments at 60°C showed that Ru@C-Al_2_*O*_3_ catalyst in aqueous media retained its activity in seven consecutive runs, indicating a stable catalytic system. The apparent activation energy of Ru@C-Al_2_*O*_3_ catalyst was calculated and amounts to 34.66 kJ/mol. Ru/γ-Al_2_*O*_3_ catalysts in the aqueous-phase hydrogenation of LA suffer from mediocre activities and stabilities mainly due to the non-uniform distribution of ruthenium on the support and because γ-Al_2_*O*_3_ is unstable in water (due to the existence of surface hydroxyl moieties) and tends after rehydration to form γ-AlOOH, i.e., boehmite. Tan et al. (2016) prepared functionalized γ-alumina supports such as NH_2_-γ-Al_2_*O*_3_ to obtain highly dispersed ruthenium nanoparticles (Figure 7) and applied them as catalytic systems in the hydrogenation reaction of LA selectively to GVL in aqueous media. Ru/NH_2_-γ-Al_2_*O*_3_ catalysts exhibited superior activity and stability compared with their Ru/γ-Al_2_*O*_3_ counterparts. For example, at 70°C with Ru/NH_2_-γ-Al_2_*O*_3_ catalysts, a TOF value of 3,355 per hour was obtained in the aqueous-phase hydrogenation of LA, whereas with Ru/γ-Al_2_*O*_3_ catalysts the activity was very lower, namely 432 TOFs per hour under the same reaction conditions. In ten recycling experiments the Ru/NH_2_-γ-Al_2_*O*_3_ catalyst retained its activity albeit the reaction temperature was high i.e., 130°C. Raspolli Galletti et al. (2012) investigated the hydrogenation of LA to GVL catalyzed by commercial 5 wt.% Ru/Al_2_*O*_3_ or 5 wt.% Ru/C catalysts combined with an acid co-catalyst such as the ion exchange resins of the type Amberlyst A70 and Amberlyst A15, niobium phosphate, and niobium oxide in aqueous media. The catalytic system 5 wt.% Ru/C-Amberlyst A70 exhibited the highest catalytic activity (TOF = 558 h^−1^) at 70°C under 5 bar dihydrogen pressure within 3 h, whereas with the 5 wt.% Ru/C catalyst in the absence of the acid co-catalyst the activity was rather low (TOF = 74 h^−1^) under the same reaction conditions. Piskun et al. (2016) have screened a series of 1 wt.% ruthenium catalysts supported on carbon, carbon nanotubes, Al_2_*O*_3_, SiO_2_, TiO_2_, ZrO_2_, Nb_2_O_5_, and zeolite β-12.5 at 90°C, 45 bar dihydrogen in aqueous media. Ru/β-12.5 catalysts exhibited by far higher catalytic activities even with a factor of 5 in comparison to the other ruthenium-supported catalysts, to obtain a conversion of LA of 94 mol% and selectivity to GVL, and 4-hydroxyvaleric acid of 66 and 34 mol%, respectively, within 2 h of reaction time in water. Zhang et al. (2017) applied highly stable Ru/ZSM-5 catalysts in the aqueous-phase hydrogenation of LA to GVL at a low reaction temperature of 70°C and performed recycling experiments with 10 consecutive runs without any loss of catalytic activity indicating no deactivation of the catalyst which is mainly caused by ruthenium aggregation, leaching and carbon deposition on the catalytically active sites. It has been found that a strong ruthenium-support interaction takes place at a higher tetrahedral–coordinated framework Al content, which minimizes the ruthenium aggregation and leaching under the acidic reaction conditions. Highly dispersed ruthenium nanoparticles consisting of Ru(0) to 59% and of RuO_2_ to 41%, with an average particle size of 2.3 nm on N-doped carbon nanospheres (Ru/NCS), were used as highly active catalysts (TOF= 9,858 h^−1^) in the hydrogenation of LA to GVL (Table 1) in aqueous media (Liu et al., 2019). The incorporation of N-dopant in the carbon matrix of the support plays a crucial role in the high dispersion of ruthenium nanoparticles because ruthenium nanoparticle catalysts in the absence of N-doping on carbon nanospheres (Ru/CS) were less active (TOF = 6,985 h^−1^) and displayed a clear sintering of ruthenium nanoparticles in the aqueous solvent (Table 1). Ndolomingo and Meijboom (2019) immobilized ruthenium, palladium, copper, and chromium nanoparticles with a mean particle size of 2–6 nm on mesoporous metal oxides such as TiO_2_, MnO_2_, and NiO, and applied them as catalysts in the LA hydrogenation reaction in aqueous media. At a reaction temperature of 150°C under 20 bar H_2_ within 5 h, the highest activity (TOF = 101 h^−1^) was obtained by Ru/TiO_2_ or Ru/MnO_2_ catalysts (Table 1) compared to Ru/NiO systems. Under the same conditions the reactivity exhibited Cu/TiO_2_ catalyst was rather low (TOF = 64 h^−1^). The catalytic activity of immobilized metal nanoparticles follows the order: Ru ≈ Pd > Cu > Cr and was revealed to be higher in water compared to the solvent-free system. Ruthenium nanoparticles with a mean size of 1.4 nm were embedded on dendrimers, followed by immobilization of the whole system on mesoporous TiO_2_ supports and then applied as catalysts in the LA hydrogenation reaction in the aqueous solvent in order to obtain LA conversions of 92 mol% with selectivities toward GVL and 2-MTHF of 98 and 2 mol%, respectively, and in organic solvents such as 1,4-dioxane where the conversion of LA was slightly higher (98 mol%) with selectivities to GVL and 2-MTHF of 99 and 1 mol%, respectively, at a reaction temperature of 150°C under 10 bar H_2_ pressure within 5 h of reaction duration (Nenamashi et al., 2018). Alginates which are polysaccharides in cell walls of macro algae were used to synthesize high surface area TiO_2_ and binary TiO_2_/ZrO_2_ supports for the immobilization of ruthenium nanoparticles applied as catalysts in the hydrogenation of LA toward GVL in aqueous media (Ruppert et al., 2019). The best catalytic performance was obtained with ruthenium nanoparticles possessing a bi-modal particle size distribution consisting of a mean particle size of 1.9 nm and an average size of 3.7 nm on bare TiO_2_ support, to achieve a conversion of LA of 79 mol% with a yield to GVL of 76 mol% under mild reaction conditions namely 30°C, and 50 bar of dihydrogen within 1 h of reaction time. Ruthenium nanoparticles with an average size of 2.8 nm diameter were encapsulated in sulfonated exchange resins such as DOWEX, and applied as bifunctional catalysts in the aqueous phase hydrogenation of LA to GVL under batch conditions to obtain an activity in the order of 102 TOF's per hour, with a conversion of LA of 98.3 mol% at 70°C, 10 bar H_2_ within 4 h of reaction time, and under continuous conditions at 70°C with dihydrogen pressures between 48 and 70 bar and contact times of 62–211s to observe conversions of LA between 89 and 100 mol% and excellent durability of the bifunctional Ru@ DOWEX catalyst (Moreno-Marrodan and Barbaro, 2014). The surfactant hexadecyl(2-hydroxyethyl)dimethylammonium dihydrogen phosphate (HHDMA) was used as a water-soluble ligand to modify ruthenium nanoparticles with an average diameter of 1.3 nm on TiSi_2_O_6_ support and applied as catalyst in the continuous hydrogenation of LA in water at 100–150°C, under pressures between atmospheric and up to 60 bar of H_2_ with contact times of 4–16s to obtain selectivities toward GVL higher than 95 mol%, with the selectivity to 1,4-PDO to be in the range of 5 mol% without any deactivation of the catalyst for a period of 15 h (Albani et al., 2017). A comparison to a conventional Ru/C catalyst with an average ruthenium particle diameter of 1.5 nm has shown that the Ru/HHDMA/TiSi_2_O_6_ catalytic system exhibited four times higher activity compared with the benchmark Ru/C catalyst which even suffers from severe deactivation due to formation of RuO_2_. Energy dispersive X-ray (EDX) maps of ruthenium and phosphorus in the Ru/HHDMA/TiSi_2_O_6_ system have shown that P is located in the areas where also Ru appears, and therefore it is highly probable that the HHDMA ligand is bounded to ruthenium nanoparticles with the phosphate moiety and to the TiSi_2_O_6_ carrier by the NCH_2_CH_2_OH functionality. The authors rationalized the results regarding the difference in stability of 0.24wt.% Ru/HHDMA/TiSi_2_O_6_ and 5wt% Ru/C catalysts by assuming that in the 0.24wt.% Ru/HHDMA/TiSi_2_O_6_ catalyst the phosphate groups of the ligands (ratio of HHDMA/RuNP ~ 250) shield the ruthenium nanoparticles and make difficult the oxygen approach, and also that the ligand-ruthenium interfacial acidity protects ruthenium nanoparticles from the oxidation reaction to form RuO_2_. Furthermore, DFT calculations revealed that these HHDMA ligand-ruthenium interfacial acidic properties in the highly polar aqueous solvent make it possible that the Ru/HHDMA/TiSi_2_O_6_-catalyzed reaction follows a mechanistic path with low energy barriers, resulting in a fourfold increase in catalytic activity.

#### Heterogeneous Water-Dispersible Catalytic Nanoparticles

Compared to the widely applied heterogeneous transition metal(0) catalytic nanoparticles (NPs) immobilized on the surface of various solid supports, the application of heterogeneous water-dispersible NPs catalysts constitutes a relatively novel but emerging approach in the field of aqueous-phase catalytic conversions of renewable biomass-derived platform chemicals. The aqueous solvent provides for a higher dispersion of the water-dispersible NPs able to exhibit great catalytic activities and impressive stabilities depending on the nature of the transition metal and especially on the choice of an appropriate stabilizer which plays a key role in keeping NPs with small particle size diameters avoiding aggregation under the reaction conditions, and thus keeping a large surface area due to the high ratio of surface metal atoms to bulk metal loading, which makes available a large number of catalytic active sites to convert the starting material. Consequently, a broad spectrum of various types of NPs stabilizers have been applied in different catalytic reactions which include, *inter alia*, surfactants, polymers, dendrimers, and also phosphines or nitrogen-containing ligands which are typically applied to modify homogeneous catalytic complexes (Roucoux et al., 2002; Yan et al., 2010; Dykeman et al., 2012; Yuan et al., 2012; Bouriazos et al., 2014; Bulut et al., 2015; Duan et al., 2015). Similar to the homogeneous water-soluble catalytic molecular complexes, the cumbersome separation and recycling of water-dispersible catalytic NPs in the aqueous solvent could be achieved by extraction after external addition of an organic solvent to create a biphasic system, followed by separation of the two phases.

In 2016, Patil and Bhanage studied the hydrogenation reaction of LA catalyzed by water-dispersible Ru(0) nanoparticles (RuNPs) formed *in situ* from RuCl_3_·3H_2_O precursor and polyethylene glycol (PEG) 400, used both as solvent and stabilizer at 110°C within 4 h reaction time in a solvent mixture PEG400/Water of a volume ratio 70/30. They reported a catalytic activity of TOF= 10 h^−1^ with a conversion of LA and selectivity to GVL of 99 mol%. At a higher temperature of 130°C the catalytic activity increased up to TOF= 40 h^−1^ with 99 mol% of both conversion of LA and selectivity toward GVL (Table 1). The formation of ruthenium nanoparticles was confirmed by TEM analysis and found nanoparticles of a very large size, i.e., 100–200 nm. Recycling experiments of the RuCl_3_·3H_2_O/PEG400/H_2_O system from the monophasic hydrogenation reaction mixture, followed by recovery of the catalyst by extraction and phase separation of a biphasic system created after addition of diethyl ether, have shown that the catalyst is stable without losing its activity and selectivity for six successive runs. Lower catalytic activities have been obtained in the absence of water using PEG400 alone or various organic solvents such as toluene, 1,4-dioxane, and ethanol. In, 2013 Ortiz-Cervantes and García applied water-dispersible RuNPs with a mean size of 2.4 nm as catalysts in the hydrogenation of LA prepared *in situ* from Ru_3_(CO)_12_ precursors in the absence of stabilizers. This exhibited a catalytic activity of TOF = 143 h^−1^ with a quantitative conversion of LA and selectivity to GVL at 130°C in aqueous media (Table 1). The 1,4-PDO product was obtained with a selectivity of 4 mol% at a higher temperature and pressure (150°C, 35 bar H_2_) and longer reaction time (24 h), with the selectivity of GVL to remain 96 mol% at a quantitative conversion of LA. Using RuNPs in organic solvents such as THF, the catalytic activities were lower with those obtained in water, and in the presence of alcohols such as methanol and propanol the main products were their corresponding levulinate esters. Tay et al. (2016) investigated hydrogenation reaction of LA to GVL catalyzed by water-dispersible RuNPs obtained *in situ* from both monodentate and bidentate *p*-cymene ruthenium(II) N-heterocyclic carbene (RuNHC) complexes as catalyst precursors in aqueous media. *p*-cymene RuNHC catalyst precursors with monodentate N-heterocyclic carbene ligands form RuNPS in both water and organic solvents and exhibited much higher catalytic activities in water (TOF = 361 h^−1^) compared with those obtained in organic solvents such as THF (TOF= 3 h^−1^), methanol, and isopropanol (Table 1). However, in organic solvents, *p*-cymene RuNHC catalyst precursors with bidentate N-heterocyclic carbene ligands form stable homogeneous complexes without any formation of RuNPs under hydrogenation reaction conditions and showed moderate catalytic activities. In 2017, Chen et al. synthesized water-dispersible RuNPs on cross-linked β-cyclodextrin with polyethylene glycol diglycidyl ether (PEG-CD) stabilizers and applied them as catalysts in the hydrogenation of LA to GVL in aqueous media. Ru@PEG-CD catalysts with an average particle size of 1.7 nm exhibited slightly higher activity compared to RuNPs with polyvinyl pyrrolidone (PVP), one of the most applied stabilizers, with a mean size of 2.3 nm at 80°C in water (Table 1). However, the Ru@PEG-CD catalytic system could be recycled in four consecutive runs without losing its activity, whereas the Ru@PVP catalyst already suffered from serious deactivation in the second recycling run. Protsenko et al. (2016, 2017, 2018) prepared RuO_2_ nanoparticles on hypercrosslinked polystyrene stabilizers containing amino groups (5% Ru/MN100) and on stabilizers without functionalities (5% Ru/MN270), and systematically studied the influence of operating reaction parameters on the catalytic activity of both systems applied in the hydrogenation reaction of LA selectively to GVL under mild conditions in aqueous media. It was found that 5 wt.% Ru/MN100 catalysts exhibited the highest activity (conversion of LA: 85 mol%), which was even superior to the activities obtained with commercial 5 wt.% Ru/C catalysts (conversion of LA: 64 mol%) under the same conditions (90°C, 20 bar H_2_, 50 min) in aqueous media, albeit the surface of Ru/C catalyst consists to 12.5% of Ru(0) and to 87.5% of RuO_2_ with a different degree of hydration, whereas the composition of the surface of Ru/MN100 catalyst consists to 100% of RuO_2_ with different degrees of hydration. This is remarkable because Ru(0) catalysts usually exhibit much higher activities compared to RuO_2_ in the LA hydrogenation reaction in aqueous media. Kubo et al. (2016) prepared water-dispersible boronate nanoparticles coated by polyethyleneimine (BNP) with an average diameter of 121 nm and used as support materials for ruthenium, palladium, and platinum nanoparticles with mean diameters <1 nm to catalyze the hydrogenation reaction of LA in aqueous media under mild conditions (100°C, 8 bar H_2_, 4 h). The highest conversion of LA (94.6 mol%) was obtained with Ru/BNP catalyst, which was stable in four recycling runs, whereas Pt/BNP and Pd/BNP catalysts exhibited lower activities to give conversions of LA of 46.9 and 24.4 mol%, respectively, with essentially quantitative selectivity toward GVL with all three catalysts. The authors further applied Ru/BNP catalysts in the LA hydrogenation reaction in organic media such as ethanol and toluene with however much lower activities in both solvents in comparison to water. RuNPs with an average size of 3.0 nm randomly distributed on the surface of a cross-linked sulfonated polyethersulfone (SPES) support were used as catalysts for the hydrogenation of LA to afford GVL in quantitative selectivity with a conversion of LA up to 87.9 mol% at 70°C under 30 bar H_2_ within 2 h in aqueous medium (Yao et al., 2014). The swelling properties of SPES support in water facilitated both the adsorption of LA to ruthenium catalytic active sites for its hydrogenation to yield 4-hydroxyvaleric acid intermediate, and to access easily the –SO_3_H acid sites for its dehydration reaction to give by intramolecular esterification the GVL product.

#### Homogeneous Water-Soluble Catalytic Complexes

**Figure 8:**
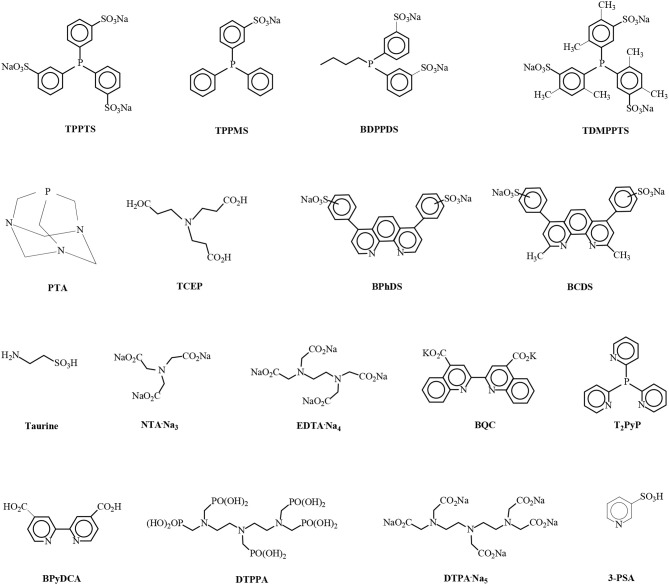
Structures of the water-soluble ligands triphenylphosphinetrisulfonic acid trisodium salt (TPPTS), triphenylphosphinemonosulfonic acid monosodium salt (TPPMS), *n*-butyldiphenylphosphinedisulfonic acid disodium salt (BDPPDS), tris(2,4-dimethylphenyl)phosphinetrisulfonic acid trisodium salt (TDMPPTS), 1,3,5-triaza-7-phosphaadamantane (PTA), tris(2-carboxyethyl)phosphine (TCEP), bathophenanthrolinedisulfonic acid disodium salt (BPhDS), bathocuproinedisulfonic acid disodium salt (BCDS), 2-aminoethanesulfonic acid (Taurine), nitrilotriacetic acid trisodium salt (NTA·Na_3_), ethylenediaminetetraacetic acid tetrasodium salt (EDTA·Na_4_), 2,2′-biquinoline-4,4′dicarboxylic acid dipotassium salt (BQC), tris(2-pyridyl)phosphine (T_2_PyP), N,N′-2,2′-bipyridine-4,4′-dicarboxylic acid (BPyDCA), diethylenetriaminepentakis(methylphosphonic acid) (DTPPA), diethylenetriaminepentaacetic acid pentasodium salt (DTPA·Na_5_) and 3-pyridinesulfonic acid (3-PSA).

The use of homogeneous water-soluble transition metal catalytic complexes in aqueous media is of great interest, particularly in the aqueous/organic biphasic mode, owing to the possibility for the heterogenization of homogeneous catalysis, which combines many advantages: (i) high activities and selectivities even under mild conditions by fine tuning of the coordination sphere of the transition metal employing a wide spectrum of water-soluble ligands in the aqueous solvent (Papadogianakis and Sheldon, 1996), (ii) easy and quantitative recovery of the catalyst in active form from organic reaction products by simple phase separation of the biphasic system and facile catalyst recycling. Consequently, numerous steps in classical homogeneous industrial processes are rendered superfluous and process engineering is enormously simplified, resulting in substantial energy savings and lower emissions (Papadogianakis and Sheldon, 1997) and (iii) new types of catalytic reactivities have been observed in the aqueous medium. The catalytic activities were much higher in water compared to organic solvents in various and different types of catalytic reactions such as hydrogenation (Moustani et al., 2018), hydrocarboxylation (Papadogianakis et al., 1997), and hydroformylation reactions (Fremy et al., 1995), which contrasts with the general perception that aqueous-phase catalysis normally exhibits lower rates compared to analogous catalytic reactions in organic solvents. Water-soluble rhodium catalytic complexes with trisulfonated triphenylphosphine ligands (TPPTS, Figure 8) have found important industrial applications such as in the Ruhrchemie/Rhône-Poulenc process for the hydroformylation of the lower olefins propylene and 1-butene, which is rich in the raffinate II mixture [raffinate II consists of 1-butene, 2-butenes (*cis*/*trans*) and butanes (*n*-/*iso*-) obtained from C_4_-stream of naphtha crackers] and in the Rhône-Poulenc process for the synthesis of vitamin E and A intermediates in aqueous/organic two-phase systems. Moreover, water-soluble palladium and rhodium complexes with monosulfonated triphenylphosphine ligands (TPPMS, Figure 8) have been used as catalysts in the aqueous/organic biphasic system in the Kuraray process, which is a four step process for the hydrodimerization of 1,3-butadiene to produce 1,9-non-anediol and 1-octanol as well (Papadogianakis et al., 1997). The hydrogenation reaction of LA in the aqueous solvent catalyzed by water-soluble ruthenium complexes proceeds in a monophasic system mode because LA is a polar and hydrophilic starting material. Therefore, the separation of water-soluble ruthenium catalytic complexes from aqueous one-phase LA hydrogenation reaction mixture proceeds with biphasic recovery of the catalyst in active form from organic reaction products by extraction and simple phase separation of an aqueous/organic two-phase system created after external addition of an organic solvent such as ethyl acetate or diethyl ether, which finally results in a facile recycling of the water-soluble catalyst.

The advent of aqueous-phase LA hydrogenation reactions catalyzed by homogeneous water-soluble ruthenium complexes could be traced back to 1977 with the pioneering work of F. Joó on hydrogenate keto carboxylic acids, such as pyruvic acid and LA by water-soluble Ru/TPPMS catalytic complexes in aqueous monophasic systems (Joó et al., 1977). Mehdi et al. (2008) reported the aqueous monophasic hydrogenation of LA catalyzed by water-soluble Ru(acac)_3_/TPPTS complexes at 140°C under 69 bar hydrogen pressure at molar ratios of LA/Ru = 600 and TPPTS/Ru = 10 within 12 h to yield 95% GVL, which was isolated after extraction by means of a biphasic system created by external addition of ethyl acetate (Mika et al., 2015). In Chalid et al. (2011) studied the biphasic hydrogenation of LA to GVL catalyzed by water-soluble RuCl_3_·3H_2_O/TPPTS systems at molar ratios of LA/Ru= 100 and TPPTS/Ru = 1, a pH value of 7, a reaction temperature of 90°C under 45 bar hydrogen pressure within 1 h to observe a conversion of 82% of LA in a dichloromethane/water (volume ratio = 100/25) two-phase system which could allow recovery and recycling of the catalyst. In a recycling experiment, however, the RuCl_3_·3H_2_O/TPPTS catalyst was partially deactivated because in the first run the conversion of LA was 81% and in the followed catalyst recycling experiment only 55% of LA was converted. Tukacs et al. (2012) described the hydrogenation of LA catalyzed by Ru(acac)_3_ modified with several water-soluble sulfonated alkylphenylphosphines under solvent-free conditions and found that the Ru(acac)_3_ precursor modified with the disulfonated diphenylbutylphosphine ligand (BDPPDS, Figure 8) exhibited the highest catalytic activity of 3,540 TOFs per hour under 100 bar hydrogen pressure at 140°C and molar ratios of LA/Ru = 6,370 and P/Ru = 10 within 1.8 h reaction time to yield 99.9% GVL. Recycling experiments of the Ru/BDPPDS catalyst were carried out using the technique of separation by distillation of volatile compounds under reduced pressure from homogeneous catalyst and addition of a new portion of LA without a significant decrease in catalytic activity for six consecutive runs. Delhomme et al. (2013) disclosed the hydrogenation reaction of LA in aqueous monophasic systems using RuCl_3_·3H_2_O and Ru(acac)_3_ catalyst precursors modified with water-soluble phosphine ligands such as TPPTS, TPPMS, trisulfonated tris(2,4-dimethylphenyl)phosphine (TDMPPTS), 1,3,5-triaza-7-phosphaadamantane (PTA) and tris(2-carboxyethyl)phosphine (TCEP) (Figure 8) at 140°C under 50 bar of dihydrogen pressure, molar ratios of LA/Ru = 100–600 and of P/Ru = 3.25–10 within 5 h reaction time. The highest activity of TOF = 210 h^−1^ was exhibited by the RuCl_3_·3H_2_O/TPPTS catalytic system, whereas with the Ru(acac)_3_/TPPTS system the activity was lower to achieve 202 TOF's per hour, and the conversion of LA and selectivity to GVL were 99 and 97 mol%, respectively. Moustani et al. (2018) investigated the hydrogenation reaction of LA using RuCl_3_·3H_2_O, Ru(NO)(OAc)_3_, Ru(NO)(NO_3_)_3_, Ru(acac)_3_, [Ru(NO)]_2_(SO_4_)_3_, and RuO_2_·H_2_O catalyst precursors modified with water-soluble phosphine and especially with nitrogen-containinig ligands such as TPPTS, PTA, bathophenanthrolinedisulfonic acid disodium salt (BPhDS), bathocuproinedisulfonic acid disodium salt (BCDS), 2-aminoethanesulfonic acid (taurine), nitrilotriacetic acid trisodium salt (NTA·Na_3_), ethylenediaminetetraacetic acid tetrasodium salt (EDTA·Na_4_), 2,2'-biquinoline-4,4'dicarboxylic acid dipotassium salt (BQC), tris(2-pyridyl)phosphine (T_2_PyP), N,N'-2,2'-bipyridine-4,4'-dicarboxylic acid (BPyDCA), diethylenetriaminepentakis(methylphosphonic acid) (DTPPA), diethylenetriaminepentaacetic acid pentasodium salt (DTPA·Na_5_) and 3-pyridinesulfonic acid (3-PSA) (Figure 8) in the aqueous monophasic system. The highest activity of TOF = 3,000 h^−1^ was obtained with RuCl_3_·3H_2_O/BPhDS catalysts at 140°C, 80 bar H_2_ pressure within 1 h and molar ratios of LA/Ru = 3,000 and BPhDS/Ru=1 with addition of 5 ml of aqueous solvent by a ruthenium concentration of 75 ppm and pH value of 2.43 where the conversion of LA was quantitative with essentially quantitative selectivity to GVL of 99.9 mol% and formation of only 0.1 mol % of the 1,4-PDO byproduct. The apparent activation energy of the Ru/BPhDS catalytic system amounts a relative low value of 53.3 kJ/mol, which is remarkable when one considers that this catalyst reduces a less reactive keto functionality into an alcohol group. Recycling experiments of the Ru/BPhDS catalyst from the aqueous monophasic LA hydrogenation reaction mixture, followed by biphasic recovery of the catalyst in active form from organic reaction products by extraction and simple phase separation of an aqueous/organic two-phase system created after external addition of diethyl ether, has shown that the catalyst is stable without loss of activity and selectivity in a consecutive run. The presence of organic solvents gives rise to a dramatic drop in catalytic activities which was obvious during the recycling experiments of the Ru/BPhDS catalytic system. After the hydrogenation run it is crucial to add small amounts of the organic solvent, i.e., only up to 10 ml of diethyl ether to the aqueous monophasic reaction mixture (10 ml), to form a biphasic system for the recovery and recycling of the Ru/BPhDS catalyst. In the case that higher quantities of diethyl ether were added for the extraction, the catalytic activity exponentially decreased in the consecutive LA hydrogenation run due to the minor amount of diethyl ether which was inevitably dissolved in the aqueous catalyst solution.

### One-Pot Hydrogenation of LA Into VA, 1,4-PDO, 2-MTHF, 2-Pentanol and 2-Butanol

**Figure 9:**
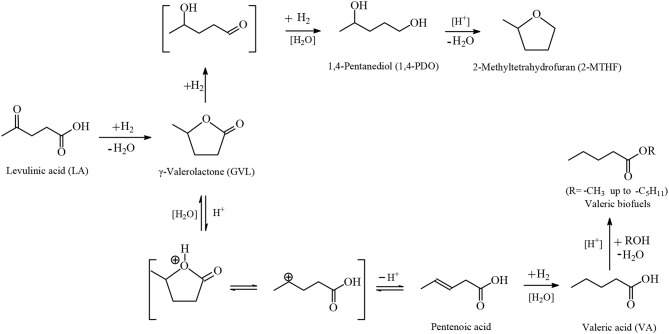
Hydrogenation of LA beyond GVL to yield VA for the production of valeric biofuels and 1,4-PDO followed by dehydration to form 2-MTHF.

**Figure 10:**
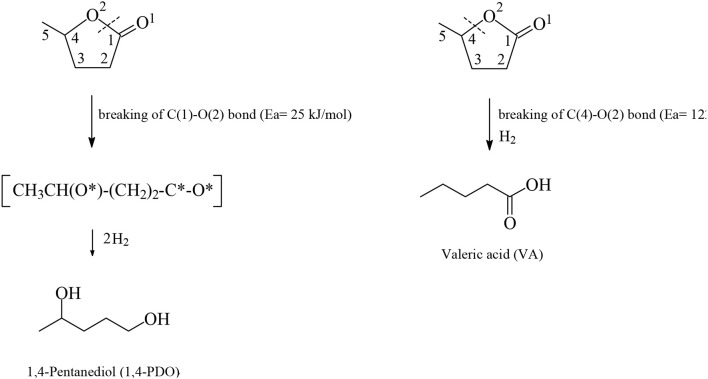
Ring-opening pathways of GVL through breaking the C(1)-(O)2 bond which after hydrogenation eventually leads into 1,4-PDO and through C(4)-(O)2 bond breaking into VA.

The catalytic hydrogenation of LA beyond GVL to yield VA, which in the presence of alcohols under acidic conditions forms alkyl valerate biofuels and 1,4-PDO followed by dehydration to obtain 2-MTHF (Figure 9), is a difficult reaction which usually takes place in the gas-phase often in the presence of platinum-based heterogeneous catalysts under severe conditions i.e., high temperatures and/or high dihydrogen pressures. For example, Kon et al. (2014) described how the one-pot hydrogenation of LA catalyzed by Pt/H-ZSM-5 at 200°C under solvent-free conditions yielded up to 99 mol% VA. Whereas, with methanol up to 87 mol% methyl valerate, Lange et al. (2010) disclosed the one-step conversion of GVL into valeric esters over Pt/TiO_2_ catalysts with a selectivity of 20–50 mol% pentyl valerate at higher temperatures of 275–300°C. Ruthenium-catalyzed one-pot hydrogenation reactions of LA into VA and 1,4-PDO are rare probably because ruthenium-based catalysts in the gas-phase suffer from drawbacks such as poor hydrogenation activity compared to analogous platinum and other transition metals-based counterparts applied in the gas-phase. The first example of one-pot hydrogenation reaction of LA into VA was reported by Luo et al. (2013), and deals with the application of the Ru/H-ZSM catalyst which contains strongly acidic sites able to catalyze the most difficult step in that sequence which is considered to be the rate-limiting step, namely the ring-opening reaction of GVL to yield at 200°C under 40 bar of dihydrogen pressure up to 45.8 mol% VA together with alkyl valerate co-products. The Ru/H-ZSM catalyst suffers from gradual deactivation, which could be attributed to dealumination resulting in a loss of catalytically active acid sites. At the higher temperature of 200°C the acid-catalyzed dehydration reaction of LA takes place with formation of α-angelica lactone, which polymerizes on the acidic sites to yield coke that after deposition also deactivates the Ru/H-ZSM catalyst. Bababrik et al. (2017) disclosed DFT calculations to present in detail the reaction mechanism of GVL conversion on Ru(0001) surfaces. The authors have shown that the GVL ring-opening step through breaking the C(1)-O(2) bond (Figure 10) proceeds rather easily with an activation energy of 25 kJ/mol and exothermicity (-31 kJ/mol), and the rate-limiting step is the hydrogenation step of the formed acyl intermediate CH_3_CH(O^*^)-(CH_2_)_2_-C^*^-O^*^ with an activation barrier of 146 kJ/mol and endothermicity (+74 kJ/mol) to yield 1,4-PDO. In contrast, the alternative pathway of GVL ring-opening through breaking the C(4)-O(2) bond (Figure 10) proceeds with more difficulty with a much higher energy barrier of 122 kJ/mol, which leads to VA (Rozenblit et al., 2016). Aqueous-phase hydrogenations of GVL catalyzed by 5 wt.% Ru/C were carried out by Rozenblit et al. (2016) at 200°C under 69 bar dihydrogen pressure to obtain a conversion of GVL of 10.3 mol% and a spectrum of products with a yield of 4.95 mol% 2-butanol, formed after C-C bond cleavage in a decarbonylation step of the surface acyl intermediate CH_3_CH(O^*^)-(CH_2_)_2_-C^*^-O^*^ obtained after GVL ring-opening, and further 4.63 mol% 1,4-PDO after hydrogenation of the acyl intermediate, 0.64 mol% 2-MTHF after dehydration of 1,4-PDO and cyclization, and 0.08 mol% 2-pentanol obtained after deoxygenation and further hydrogenation of the surface CH_3_CH(O^*^)-(CH_2_)_2_-C^*^-O^*^ acyl intermediate. Cui et al. (2018) studied the continuous one-pot aqueous-phase hydrogenation reaction of LA catalyzed by molybdenum-modified Ru/C catalysts to obtain almost quantitative conversion of LA and selectivities of 96.7 mol% 1,4-PDO, 0.2 mol% 2-MTHF, 0.5 mol% GVL, 0.6 mol% 2-butanol and 2.0 mol% 2-pentanol using an atomic ratio of Mo/Ru= 0.25 under mild conditions at 70° C and 40 bar dihydrogen pressure. *In situ* FTIR investigations revealed that RuNPs are responsible for the activation and dissociation of the dihydrogen reactant and on Mo catalytically active species the absorption and activation of the keto functionality of LA starting material takes place. The Ru-MoO_x_/C catalyst remained stable for a period of 200 h continuous one-pot LA hydrogenation reaction toward 1,4-PDO without any deactivation. Lv et al. (2018) reported the one-pot aqueous-phase hydrogenation of LA catalyzed by nanoporous ruthenium-based catalysts to obtain at 100°C under 60 bar H_2_ within 6 h reaction time a yield of 74.6 mol% 1,4-PDO, 1.9 mol% 2-MTHF, 4.6 mol% GVL, 10.4 mol% 2-butanol and 5.0 mol% 2-pentanol. At a higher temperature of 140°C the yields of both 2-butanol and 2-pentanol were increased to 63.6 and 15.2 mol%, respectively, with, however, a low yield of 1,4-PDO of 0.9 mol%. Mizugaki et al. (2017) and Mizugaki and Kaneda (2019) investigated the one-pot hydrogenative C-C bond cleavage reaction of LA catalyzed by RuNPs with an average particle diameter of 3 nm supported on CeO_2_ to achieve almost an quantitative conversion of LA and a yield of 85 mol% 2-butanol and of 5 mol% 2-pentanol at 150°C under 30 bar dihydrogen pressure within 12 h reaction time in aqueous media. The presence of the aqueous solvent is indispensable in the Ru/CeO_2_-catalyzed hydrogenative C-C cleavage reaction of LA toward 2-butanol, because in organic solvents such as 2-propanol, THF or dimethoxyethane the yield to 2-butanol was only up to 5 mol% and the major products were GVL of 70 mol% and 1,2-PDO of 23 mol%. Licursi et al. (2018) studied a cascade process for the direct conversion of LA or GVL into 2-MTHF or 2-butanol and 2-pentanol, employing commercially available catalysts in the aqueous solvent. The authors used for the first time a catalyst combination of Ru/C along with Re/C, together with niobium phosphate to obtain selectivities toward 2-MTHF of 28 mol% or 65 mol % from LA or GVL substrates, respectively. In sharp contrast employing a catalyst combination comprising of Ru/C and HY zeolite at 200°C under 30 bar dihydrogen pressure, the reaction takes another course and yields a mixture of 2-butanol with 2-pentanol of 88.8 mol% and 100 mol% from LA and GVL starting materials, respectively, in aqueous media.

## Summary and Outlook

The catalytic hydrogenation route for the valorization of LA has received considerable interest recent years because of the wide range of potential applications of LA's hydrogenation products which include, *inter alia*, advanced biofuels, fine chemicals, solvents and additives to gasoline or to food. In the last decade the hydrogenation reactions of LA into GVL, which is a key intermediate compound, and beyond GVL to yield VA, 1,4-PDO, 2-MTHF, 2-pentanol, and 2-butanol have gained a lot of attention and various transition metal catalytic systems have been developed in the absence and presence of organic or aqueous solvents. Remarkable progress has been made, however, in the application of ruthenium-based catalytic systems which have been used extensively due to the inherent ability of ruthenium under mild conditions to hydrogenate the carbonyl functionality of levulinic acid selectively into an alcohol group to form 4-hydroxyvaleric acid intermediate, which spontaneously after dehydration and cyclization yields GVL. This critical review has summarized and discussed the progress made in the last decade in the field of aqueous-phase ruthenium-catalyzed hydrogenation reactions of LA employing heterogeneous catalysts on solid supports and heterogeneous water-dispersible catalytic nanoparticles or homogeneous water-soluble catalytic complexes with biphasic catalyst separation. The significance of the aqueous solvent to carry out catalytic hydrogenation reactions of LA has been highlighted because the presence of water combines many environmental and economic benefits. Therefore, this review constitutes a guidance for the development of novel efficient ruthenium-based catalytic systems which would be more active and especially capable for the one-pot hydrogenation of LA toward VA, 1,4-PDO, 2-MTHF, 2-pentanol and 2-butanol. At the same time it offers guidance on more stable and recyclable systems suitable as catalysts for industrial-scale LA hydrogenation reaction in green and sustainable aqueous solvents to be integrated into biorefineries of the future.

## Author Contributions

GP has written this review. All other authors have equally contributed and approved it for publication.

## Conflict of Interest

The authors declare that the research was conducted in the absence of any commercial or financial relationships that could be construed as a potential conflict of interest.
